# Optimization of Structural and Processing Parameters for Selective Laser Melting of Porous 316L Bone Scaffolds

**DOI:** 10.3390/ma15175896

**Published:** 2022-08-26

**Authors:** Shubo Xu, Sen Zhang, Guocheng Ren, Yuefei Pan, Jianing Li

**Affiliations:** 1 School of Materials Science and Engineering, Shandong Jianzhu University, Jinan 250101, China; 2 State Key Laboratory of Materials Processing and Die & Mould Technology, Huazhong University of Science and Technology, Wuhan 430074, China

**Keywords:** porous bone scaffolds, residual stress, finite element simulation, optimization

## Abstract

In the implantation of porous bone scaffolds, good mechanical properties of the scaffold are a prerequisite for the long-term functionality of the implanted scaffolds, which varies according to the structure and the forming process. In this study, the influence of the forming parameters and structure of the Selective Laser Melting (SLM) process on the mechanical properties of 316L stainless steel bone scaffolds was investigated using finite element simulation combined with experimental methods. The mechanism of the influence of the process parameters and structure on the mechanical properties of bone scaffolds was summarized using static compression finite element numerical simulations, compression experiments, hydrodynamic simulations, forming numerical simulations and SLM forming experiments. The results show that the magnitude of residual stress and the distribution of defects under different process parameters had a strong influence on the microstructure and properties of the scaffold, and the residual stress of the Body-Centered Cube (BCC) structure formed at an energy density of 41.7 J/mm^3^ was significantly reduced, with less surface spheroidization and fewer cracks on the melt pool surface. The smallest grain size of 321 nm was obtained at an energy density of 77.4 J/mm^3^, while in terms of mechanical properties, the optimization of the structure resulted in an 8.3% increase in yield strength and a reduction in stress concentration. The predictions of stress, deformation, and forming quality during construction with different process parameters, achieved using finite element analysis, are basically in agreement with the experimental results, indicating that the best process parameters for forming BCC structural supports were determined by using finite element simulation combined with experiments; moreover, the distribution and evolution of residual stresses and defects under different process parameters for constructing BCC structures were obtained.

## 1. Introduction

In the field of bone-tissue engineering, the ability of a bone scaffold to perfectly fit the mechanical properties of the host bone is one of the key research questions in regenerative medicine [1]. Natural bone is an interconnected porous structure [2], which can play an active role in cell infiltration, proliferation, and differentiation. While conventional manufacturing methods are unable to have excellent mechanical properties while precisely controlling the internal structure of the implant, the advent of additive manufacturing technology has made it possible to have materials with both material transport and biological properties [2,3]. Metals and their alloys have been studied by many authors for bone implantation [4,5], with 316L stainless steel, cobalt-based alloys and titanium, and their alloys, being widely used for their good biocompatibility and excellent corrosion resistance. The difference in the elastic modulus between natural bone and implants becomes an important influencing factor in late bone reconstruction and resorption, and the mechanical characteristics and adjustable porosity of porous scaffolds make them suitable materials for repairing or replacing damaged bone [6,7,8].

To date, many scholars have studied porous skeletal scaffolds and have made significant breakthroughs in the study of pore size, unit structure, and porosity. The relationship between the porosity, structural characteristics, and mechanical properties of typical structural units was deduced by Wang et al. [9] after a study of the mechanical properties of BCC structural scaffolds. Cuadrado et al. [10] investigated three different porous structures such as BCC and compared the stress–strain behaviour of the different porous structures using mechanical experiments. Arjunan et al. [11] conducted a general study of stiffness, strength, and stress concentration in scaffolds with different porosity and found that pore shape has an important influence on the permeability, stiffness, and strength of the bones. In terms of structure and bioactivity, Rudrich U et al. [12] found that bone cells did not grow well in right-angled and convex structures, and Van Bael S et al. [13] found that cell blockage was particularly pronounced in large-angle structures by studying the shapes of different pore structures. Good structural and mechanical properties are necessary for cell proliferation, and the quality of the scaffold is an important prerequisite for the long-term functional use of bone scaffolds. It has been found that by optimizing the process parameters of SLM machining, the generation of processing defects can be reduced, and thus, excellent mechanical properties can be achieved. Ma et al. [14] concluded that higher scanning speeds can be used to obtain uniform and fine microstructures during the forming process due to the faster solidification rate. Jiang et al. [15] and Anil et al. [16] investigated the effect of energy density on the density and defects of SLM-formed parts and found that the use of suitable energy density can result in formed parts with excellent properties. Yadollahi et al. [17] found that the construction process parameters play a decisive role in thermal cycling during the forming process, thus affecting the tissue structure and, hence, the mechanical strength under static and cyclic loading. Wang et al. [18] found that rapid solidification of the material and repeated thermal cycling can lead to the accumulation of residual stresses, which can lead to part deformation and negatively affect the mechanical properties of the part.

The existing medical research on additive manufacturing has focused on the biomechanical properties of different porosities, pore sizes and structures, and bio-clinical implantation, while some research has been conducted on the structural optimization of porous scaffolds [19,20]. The effects of different construction parameters on the defects, residual stress, and deformation of a particularly structured scaffold have not been explored in depth, and since the scanning surface of the laser is intermittent when SLM forms porous scaffolds, the discontinuity of the scanning makes it easy to introduce different degrees of stress and strain when building porous scaffolds. For these reasons, this study decided to take the BCC structure as the research object and optimize the construction process parameters based on the optimized structure; the purpose of this was to reduce the generation of defects, reduce residual stress and deformation, enhance the mechanical properties of the porous scaffold, obtain a BCC porous scaffold with excellent performance, and provide a theoretical basis for the practical application of porous structures.

## 2. Materials and Methods

### 2.1. Selection of Materials

316L stainless steel is one of the best materials for bone replacements due to its excellent corrosion resistance, mechanical properties, low price, and good processing properties, and is now widely used as a metal-based medical implant material. Therefore, in this study, 316L was used as the raw material for SLM processing, and an argon atomization method was used to produce 316L powder, with powder diameters ranging from 15–51 um and an average particle size of 27 um.

### 2.2. Choice of Experimental Protocol

In this study, it was decided that the test would be carried out using an energy density of between 40 and 120 J/mm^3^ after combining the test equipment with the research results of many scholars [15,21]. The energy density was, in turn, determined by the laser power, scanning speed, powder layer thickness, and scanning spacing, so this study decided to choose a laser power of 100 W–160 W, a scanning spacing of 0.06–0.10 mm, a scanning speed of 500–700 mm/s, and a powder layer thickness of 0.04 mm for the construction of porous skeletal scaffolds, for each factor in the study. Three different values were selected for the tests, and the test protocols are shown in Table 1.

## 3. Experimental Work

### 3.1. Structure Construction

**Figure 1:**
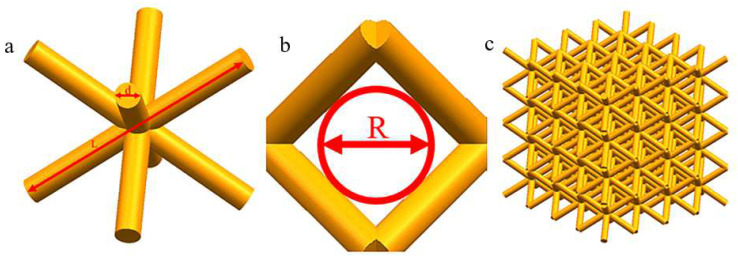
(**a**) BCC structural unit, (**b**) pore diameter of porous bone scaffold, and (**c**) finite element analysis model.

The scaffold models were generated using UG12.0 (Siemens PLM Software, Plano, TX, USA). The BCC structure [9,11,21] was chosen as the experimental object. The BCC structure unit is shown in Figure 1a. Porosity and pore size are important parameters in the design of implantable bone scaffolds, and the study used a structure with a porosity of about 85% and a pore size of 850 μm for analysis [8,22,23,24]; the porosity and pore size *R* (as shown in Figure 1b) of the scaffold were controlled by setting the cylindrical diameter *d* and the cylindrical length *l.* The specific design parameters are shown in Table 2.

### 3.2. Static Mechanics Finite Element Simulation

The study used Ansys software for the static analysis of BCC porous structures, in order to ensure that the computational accuracy was improved while saving computational resources, the model cell mesh was divided using a tetrahedral mesh with a mesh size of 0.05 mm. The static mechanical model was composed of 4 × 4 × 4 cells, as shown in Figure 1c.

The boundary conditions of the finite element model were consistent with the compression experiments, and the calculated load was an axial downward compression force of 3000 N. The boundary conditions at the bottom of the model were set to fully constrained.

### 3.3. Computational fluid Dynamics (CFD) Finite Element Simulation

**Figure 2:**
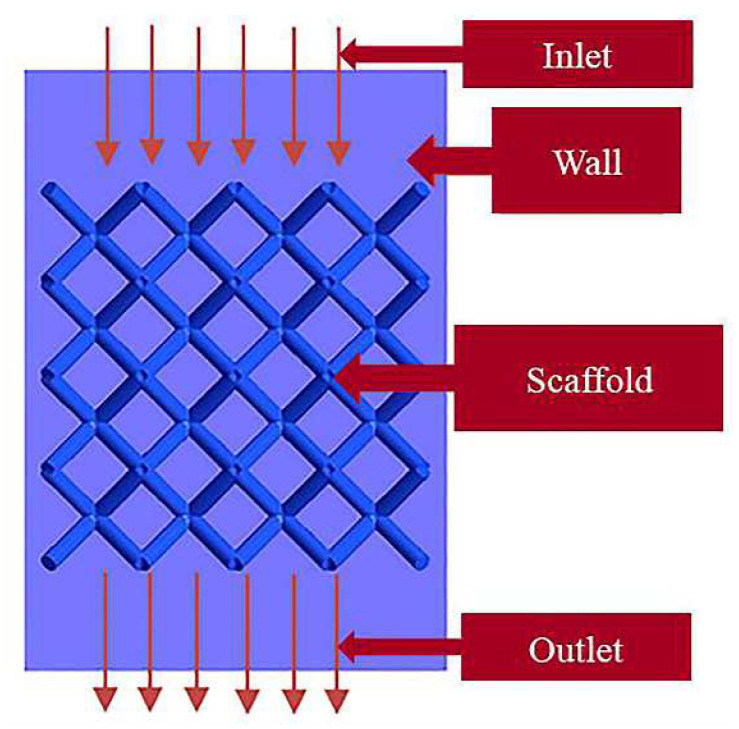
Schematic diagram of simulation boundary conditions.

The computational model was assembled using 4 × 4 × 4 units, as shown in Figure 1c. Figure 2 shows the CFD simulation boundary conditions for the porous bone scaffold. The object of analysis was an incompressible fluid with constant density, using the Navier–Stokes Equation (1) [8,25], where ρ, v and u denote the fluid density (kg/m^3^), fluid flow velocity (m/s), and dynamic fluid viscosity (kg/m/s), respectively. ∇ is the del operator, and p and F denote pressure (MPa) and force (N), respectively. The permeability K for both structures was calculated from Darcy’s law (Equation (2)) [25,26], where l is the characteristic length (mm) and ΔP is the pressure difference (MPa).

In the finite element simulation the inlet velocity was set to 1 mm/s, the inlet pressure to 0 MPa, the fluid density to 1050 kg/m^3^ and the viscosity to 0.0037 kg/m/s [2,26].

(1) $$\left\{\begin{array}{l} \rho \frac{\theta v}{\theta t}-u\nabla^{2}v+\rho \left(v\cdot \nabla \right)v+\nabla p=F\\ \nabla \cdot v=0 \end{array}\right.$$

(2) $$k=\frac{u\cdot v\cdot l}{\Delta p}$$

### 3.4. Finite Element Analysis of the Forming Process

The study used MSC Simufact Additive software for welding to simulate the forming process of porous structural scaffolds. Simufact Additive software is based on inherent strain theory for finite element calculations and is capable of analyzing the generation, evolution, and removal of internal stresses in porous structural scaffolds.

The study used MSC Simufact Additive and MSC Simufact welding software to simulate the forming process for porous structural scaffolds. The Simufact welding software allows the welding parameters and paths to be customized by modifying the system program to visualize the changes in the melt pool during the welding process. Simufact Additive software is based on inherent strain theory for finite element calcula-tions and is capable of analyzing the generation, evolution, and removal of internal stresses in porous structural scaffolds.

## 4. Results and Discussion

### 4.1. Static Mechanical Results

As the main support organ of the human body, the main role of bone is protection and support; therefore, for the implantation of scaffolds, excellent resistance to compression can effectively improve the implantation of porous scaffolds. Avoiding or reducing deformation during use and reducing stress concentrations is particularly important for the use of porous bone scaffolds.

**Figure 3:**
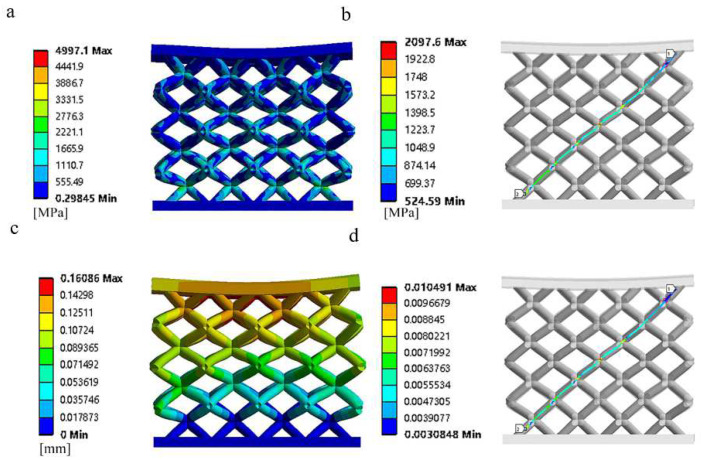
Nephogram of simulated compressive stress, strain, and deformation of bone scaffold: (**a**) finite element stress distribution nephogram, (**b**) diagonal stress distribution in bone scaffold, (**c**) finite element displacement distribution nephogram, and (**d**) diagonal strain distribution in bone scaffold.

Figure 3 shows the compression stress, strain, and deformation clouds of the simulated bone scaffold. In the finite element calculations, in order to ensure that the brace can be subjected to uniform forces, thin plates were placed on the upper and lower parts of the brace for loading compression. From Figure 3a,b, it can be seen that the stress concentration phenomenon of the BCC structure mainly appears in the diagonal intersection part of the brace’s connecting holes, with less stress on the cylindrical rod. Figure 3c,d show that the deformation of the BCC unit structure varies considerably, with the upper part of the scaffold showing a more pronounced twist and tilt than the lower part due to the load distribution above, with the deformation and strain mainly concentrated at and near the connection points.

**Figure 4:**
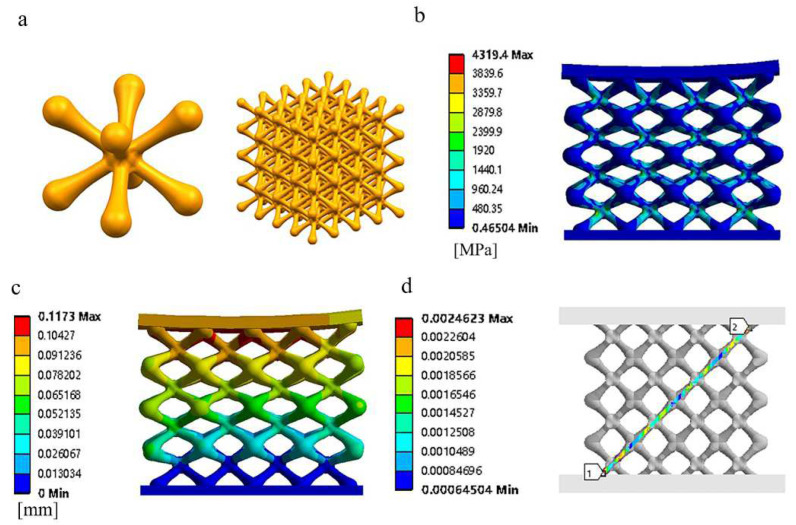
Nephogram of simulated compressive stress, strain, and deformation of bone scaffold: (**a**) optimized BCC Structure, (**b**) finite element stress distribution nephogram, (**c**) finite element displacement distribution nephogram, and (**d**) diagonal strain distribution in bone scaffold.

To address this problem, the conventional BCC structure is improved, as shown in Figure 4a. The stress concentration in the sharp-angled joint of the connecting hole is reduced using a rounded transition, and a nodal ball is added to the joint to expand the surface area for stress dispersion. At the same time, in order to compare smoothly with the conventional structure, the porosity and pore size of the optimized structural model are kept in line with the conventional structure; the specific design parameters after the adjustment of the unit column size, nodal ball size, and transition radius are shown in Table 3.

Figure 4b shows the compressive stress cloud of the optimized structure, and Figure 4c,d show the results of the deformation and strain distribution of the optimized structure. It can be seen from the figure that in the optimized structure, the addition of the nodal ball significantly improved the stress concentration magnitude, the maximum concentrated stress magnitude was reduced from the original 4997.1 MPa to 4319.4 MPa, and the average stress of the optimized structure was reduced by 14.2% compared to the conventional structure; the difference in the shape of the structure in the simulated compression was small. The maximum deformation value was reduced by 27% compared to the original and the maximum strain value was reduced by 76.5% compared to the conventional structure.

**Figure 5:**
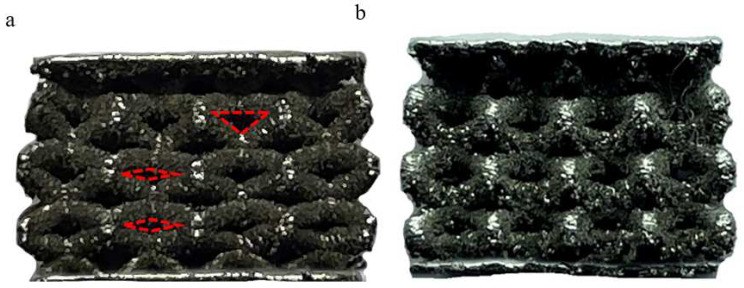
Compression test results: (**a**) conventional BCC structure and (**b**) optimized BCC structure.

Figure 5 shows the experimental compression of the two bone scaffolds, which were compressed axially using a speed of 1 mm/min and stopped when the static load force reached 3000 N. From Figure 5a it can be seen that the cylindrical rod radiating from the center of the unit body underwent significant bending deformation, a slight fracture occurred at the joints between the apertures, and the apertures underwent drastic deformation. As for the optimized BCC structure, no fracture is found at the joints, as seen in Figure 5b, and the change in the shape of the unit structure is small; however, the deformation trend of the optimized structure is similar to that of the conventional structure in terms of the bending and tensile deformation of the unit cylinder, which is, of course, due to the characteristics of the structure itself. The BCC structure is dominated by the bending deformation mechanism during compression, and continued compression may result in a ductile fracture.

The comparison shows that the static compression experiments using FEM simulations are basically the same, in terms of stress and deformation distribution, as the actual compression experiments in terms of the location of the severe deformation, with both showing tensile bending deformation of the cylindrical connecting rod. For the two different structures, the comparison clearly shows that the optimized structure has a huge advantage in terms of compressive deformation and is able to produce smaller compressive deformations with good mechanical properties.

**Figure 6:**
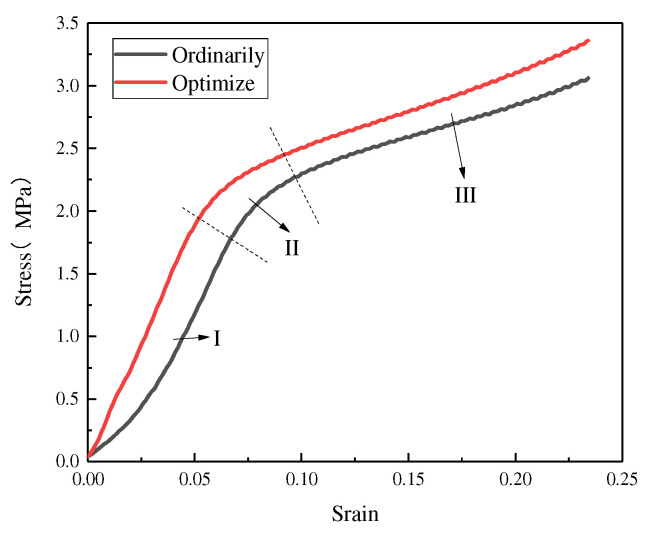
Stress–strain curve.

Figure 6 shows the stress–strain curves of the two structures, in which three regions can be defined as the deformation history of the BCC’s structure elastic loading (I), elastoplastic collapse (II), and plastic collapse (III), respectively. Combined with the analysis in Figure 5, it is concluded that at the beginning of the pressure loading is mainly elastic–plastic deformation with bending and stretching as the dominant behavior; then, as the compression increases, the length of the unit column strut is too short and cannot continue to withstand the bending, and continuous collapse occurs; this produces plastic deformation, making the scaffold tend to densify, which, in turn, improves the compressive performance. In addition, the effect of the adhesion of unmelted or semi-melted powder around the central junction of the unit body and at the intersection of the unit column should be taken into account for the third stage of the compressive performance enhancement to make the bone scaffold resistant to buckling.

Table 4 shows a comparison of the compressive strength of the two structures. It can be seen that the compressive performance of the optimized structure has been significantly improved, providing excellent performance for the implantation of the bone scaffold. In order to better adapt to the mechanical properties of human bone, the magnitude of the elastic modulus of the porous bone scaffold becomes the primary prerequisite for measuring whether the porous bone scaffold meets the implantation requirements. The Gibson–Ashby formula [27,28] is usually used as the guiding model for the calculation of the elastic modulus of porous parts, and the formula for calculating whether the porous scaffold meets the implantation requirements can be calculated according to the Gibson–Ashby model formula, as shown below.

(3) $$\frac{E^{*}}{E_{S}}=C{\left(\frac{\rho *}{\rho_{S}}\right)}^{m}$$

where E* is the elastic modulus of the porous bone scaffold, *Es* is its solid elastic modulus, *Es* = 210 GPa, ρ* is the density of the porous bone scaffold, *ρs* is its solid density, *ρs* = 7.98 g/cm^3^, and C and m are the open-structure geometric constants of *C* = 1 and m = 2, respectively. After the cleaning process of the two structures, the actual elastic moduli of the two porous bone scaffolds were measured, as shown in Table 5 shows. The modulus of elasticity of human cortical bone is 17–20 GPa and the modulus of elasticity of cancellous bone is 3.2–7.8 GPa, so both structures meet the requirements of the modulus of elasticity of human bone and can be used as cortical bone implant scaffolds.

Porous structures are designed in such a way that the fatigue limits of their microstructure are important for the proliferation of implanted cells at a later stage, and human bone, as a support scaffold for all human functions, plays an important role in the movement, support, and protection of the body. In the preparation of long-term functional skeletal scaffolds, the mechanical properties and biocompatibility of the scaffold are primary considerations, while the long-term stability of the skeletal implant has been the clinical aspect that needs to be further improved [28].

Given that both structures are prepared from the same material, it can be concluded that the increase in the stiffness of the bracket is due to the optimization of the shape, and that a certain degree of optimization of the conventional BCC structure can significantly improve the mechanical properties of the structure. In terms of optimizing the shape of the porous bone scaffolds, the optimization of conventional structures has gradually become a new research hotspot for many scholars, which provides a new reference for structural aspects of implantable bone scaffolds. Ali [29] improved the structure of Face Centered Cubic (FCC) structures and found that the scaffold became stiffer under small strains; moreover, Xiong [30] improved the functional-gradient porous structure and found that the mechanical properties of the optimized structure were significantly improved. The present study, which is at the forefront of international research to optimize the BCC structure, shows a significant reduction in stress concentration and a more desirable yield strength, which provides a new reference for structural improvements in bone implant scaffolds.

### 4.2. Hydrodynamic Results and Analysis

**Figure 7:**
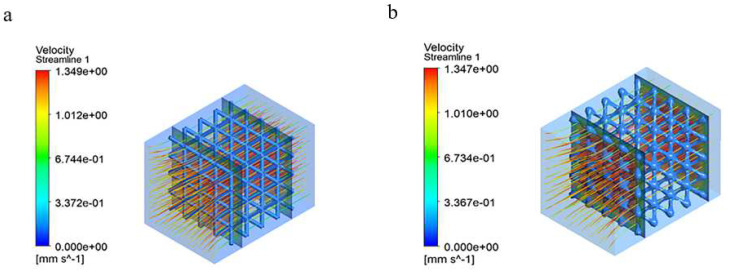
Two groups of scaffolds’ velocity clouds: (**a**) conventional BCC Structure and (**b**) optimized BCC Structure.

Figure 7 show the results of the hydrodynamic simulations. Cell aggregation and adhesion are key components of osteogenesis in porous scaffolds, and the internal permeability and fluid velocity of a bone implant scaffold have an important impact on the regeneration of bone tissue after implantation [25], where a suitable structure and good permeability inside the scaffold can provide the necessary nutrients for cell growth. Too fast a permeability or flow rate is not conducive to the adhesion of new cells to the surface of the scaffold, while too slow a flow rate may cause a large accumulation and overlap of cells at the junction and blockage within the scaffold, thus preventing the transport of nutrients. Ouyang et al. [8] showed that there was little difference in cell recruitment results between pore sizes of 650 and 850, and that the optimal flow rate for cell adhesion to the skeletal scaffold was 0.63 mm/s. Campos Marin A et al. [31] found that most cells adhered to the implanted scaffold at a flow rate of 0.5 mm/s. In conjunction with the CFD fluid simulation calculations of this study, it was found that the most suitable area for cell recruitment for both BCC structures was around the scaffold, a result that is more consistent with the actual osteoblast proliferation phenomenon [25], with a calculated permeability of 2.3 (10^−7^·m^2^) for the conventional BCC structure and 1.9 (10^−7^·m^2^) for the optimized BCC structure. It is worth noting that for the optimized BCC structure, the area suitable for cell aggregation was significantly larger, and the addition of transition nodal spheres provided a larger area for cell adhesion and regeneration.

The evaluation of the permeability of the bone scaffold using hydrodynamic simulations showed that the accelerated flow rate and low permeability of the conventional BCC structure could potentially damage the cell walls at the sharp angles of the joints and affect cell activity, while the presence of some right-angled edges in the structure itself could have a negative impact on bone cell proliferation. The addition of transitional nodal spheres in the optimized BCC structure improved the flow rate and permeability, and the more rounded angle of the joints reduced the damage to the cell attachments.

### 4.3. Bone Scaffold Forming Stress

Good mechanical properties of bone scaffolds are a prerequisite for implantation, but the selection of suitable parameters to reduce scaffold forming defects is also essential for implantation. Uneven temperature fields tend to generate thermal stresses which, in turn, generate tensile/compressive residual stresses in the melt pool, leading to cracks and, thus, affecting the quality of the scaffold forming [32,33]. This section takes the residual stresses in the scaffold as an entry point to investigate the link between residual stresses and defects such as holes and cracks, and to investigate the residual stresses and the histomorphology of the scaffold for different build energy densities to select the appropriate build parameters and to improve the quality of the forming.

**Figure 8:**
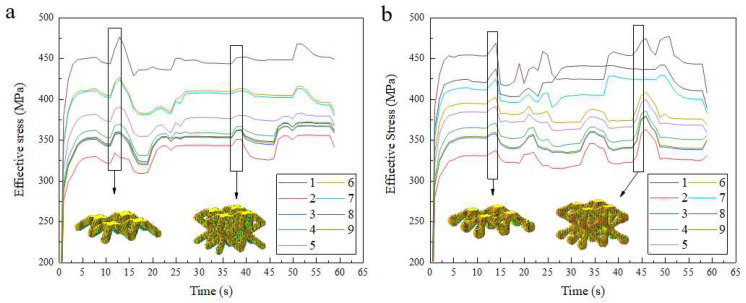
Construction stress variation diagram: (**a**) conventional BCC Structure and (**b**) optimized BCC Structure.

Figure 8 shows the variation of the instantaneous residual stresses during the construction of the bone scaffold for nine different construction process parameters. As shown in Figure 8, for both scaffold structures using experimental scenario 2, both the instantaneous residual stresses while building and the post-build residual stresses were minimal at an energy density of 41.7 J/mm^3^, in contrast to experimental scenario 8, where the build stresses and post-build stresses were consistently highest at an energy density of 111.1 J/mm^3^. As can be seen from the diagram, the sudden increase in stress inside the stent at the beginning of the construction is due to the scaffold being printed by laying powder on the substrate of the forming bin, which is at room temperature; when the first layer of powder is fused, the sharp temperature difference causes the substrate to produce uneven thermal expansion and residual deformation, which introduces a large amount of internal stress in the substrate, while the solidification layer also produces internal stress, and these resist each other in the substrate. As the build height increases, the uneven repetitive thermal cycling causes frequent thermal expansion and contraction within the substrate, introducing a large number of residual stresses, which, in turn, affect the magnitude of the stresses in the scaffold. The residual stresses inside the scaffold also increase suddenly when the central joint of the BCC structure is built, presumably because the build area increases at this time and is also relatively concentrated; this renders the solidification and shrinkage phases subject to internal stresses generated by the structure itself in addition to the resistance of the unit column below to resist shrinkage deformation. Additionally, as new layers are continuously added, the deformation is continuously blocked, introducing new tensile stresses to the new layers and introducing compressive stresses to the underlying layers, which accumulate and increase.

The damaging consequences of the presence of residual stresses are unimaginable. As one of the representatives of SLM forming, the quality of the forming of porous scaffolds is something we need to pay special attention to, especially when the bone scaffold enters the human body, which may bring unpredictable disasters; thus, avoiding or reducing the harm caused by residual stresses and extending the functional life of the bone scaffold is something we must pay special attention to. At present, the main methods used in the world to test residual stress are mechanical measurement and non-destructive measurement. It is not feasible to use the mechanical measurement for the precision structure of bone scaffold. The test method chosen for this study was X-ray diffraction for the testing of SLM-formed porous scaffolds, which is also one of the most widely used non-destructive measurement methods in the international arena.

**Figure 9:**
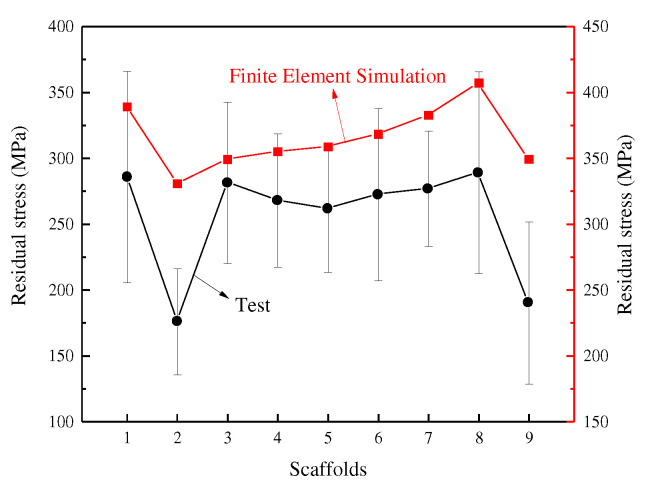
Finite Element Simulation and Experimental Test of Residual Stress.

The test location is the area in and around the connection point of the porous scaffold because the conventional structure of the scaffold connection area is small and part of the metal powder is difficult to remove, which has a greater impact on the test results; so, the test piece chosen for this study was the optimized structure. the experimental and test results are shown in Figure 9.

It can be seen from the results that there is some variation in the results obtained from the finite element simulations and the actual experimental tests, which is normal. The tests found that the residual stresses in the optimized BCC structure were compressive stresses, generally tensile stresses in the weld and its adjacent area, and compressive stresses further away from the weld. The experimental data show that the maximum value of residual stresses occurs in bone scaffold 8 and the minimum value occurs in scaffold 2. The distribution of the intermediate values is negligible and differs from the finite element simulation. The results of the finite element simulation are in general agreement with the experimental test results, which proves that the influence of the finite element simulation construction parameters on the stress of the porous scaffold can have a certain degree of significance with reference to the actual test.

**Figure 10:**
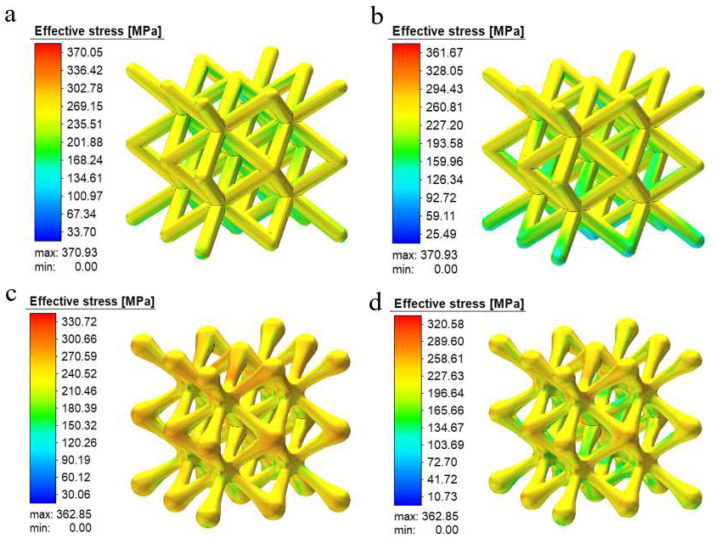
Cloud diagram of scaffold forming stress: (**a**) Before conventional structural base plate removal, (**b**) after conventional structural base plate removal, (**c**) optimization of the structure before cutting the base plate, and (**d**) optimization of the structure after cutting the base plate.

Figure 10 shows the distribution of residual stresses in the two structures. From the graph, it can be seen that the maximum stresses in the construction of both structures occur at the central joint or the intersection of the unit columns, probably because the construction area is more concentrated here, resulting in the solidification layer below the cooling and shrinking stage; this prevents the melt pool from contracting freely, generates uneven plastic deformation, and introduces residual stresses; additionally, because the thermal conductivity of the solidified area below is significantly higher than that of the surrounding powder, it results in uneven cooling of the melt pool and the formation of a large temperature gradient in the surrounding area, thus generating residual stresses.

It can also be seen in Figure 10b,d that the residual stresses inside the scaffold are significantly reduced after the two structures are cut from the substrate and the distribution of residual stresses are also reduced. The reason for this is presumably that the large temperature gradient during construction caused the substrate and the bottom of the scaffold to resist each other’s internal stresses; when the scaffold became unconfined after the scaffold was cut from the substrate, the residual stresses were released and, thus, the internal residual stresses were reduced. Another point that cannot be overlooked is that the optimized BCC structure appears to have significantly lower residual construction stresses. In combination with the concentrated stresses seen in the compression tests in the previous section, it can be seen that the nodal-ball transition method can theoretically reduce or solve residual stresses in the forming process and stress concentration defects in service.

### 4.4. Defects in Scaffold Forming

**Figure 11:**
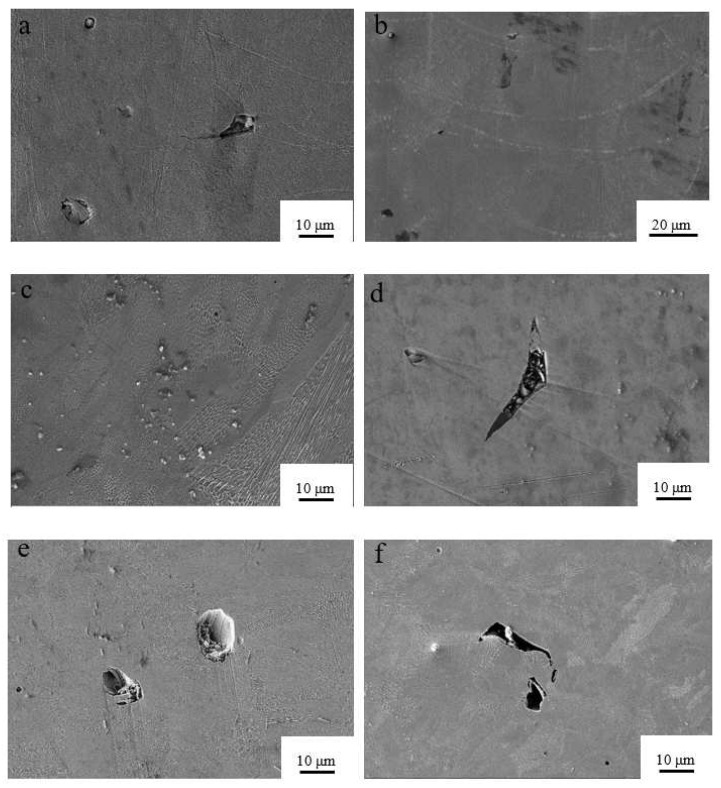

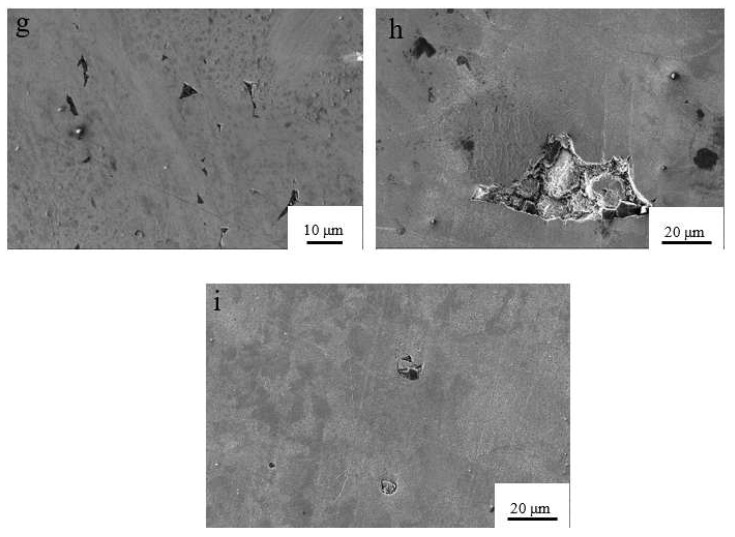
Forming quality at different energy densities: (**a**) *E* = 83.3 J/mm^3^, (**b**) *E* = 41.7 J/mm^3^, (**c**) *E* = 44.6 J/mm^3^, (**d**) *E* = 65.0 J/mm^3^, (**e**) *E* = 67.7 J/mm^3^, (**f**) *E* = 77.4 J/mm^3^, (**g**) *E* = 100.0 J/mm^3^, (**h**) *E* = 111.1 J/mm^3^, and (**i**) *E* = 57.1 J/mm^3^.

Figure 11 shows a photograph of the organization of the optimized BCC structure under different process parameters. From the figure, we can see that the defects produced at the nine energy densities are also significantly different, and we can classify the defects produced into three categories: the first category is similar to round-shaped holes, the second category is irregular, long strips loaded with holes, and the third category is residue. It is found that the main defects in the BCC structure at an energy density of <65.0 J/mm^3^ are the first and third categories, and at an energy density of ≥65.0 J/ mm^3^, the defects are mainly of the second type. Moreover, there is residual slag and semi-molten powder in the cavities, with a varying number of cracks around the holes.

The SLM forming process is a melt-solidification iteration, so in order to better investigate the causes of defects in the scaffolds, finite element simulations of the temperature and stress fields at different energy densities during the SLM forming process were carried out in this study. The results are shown in the figure below.

**Figure 12:**
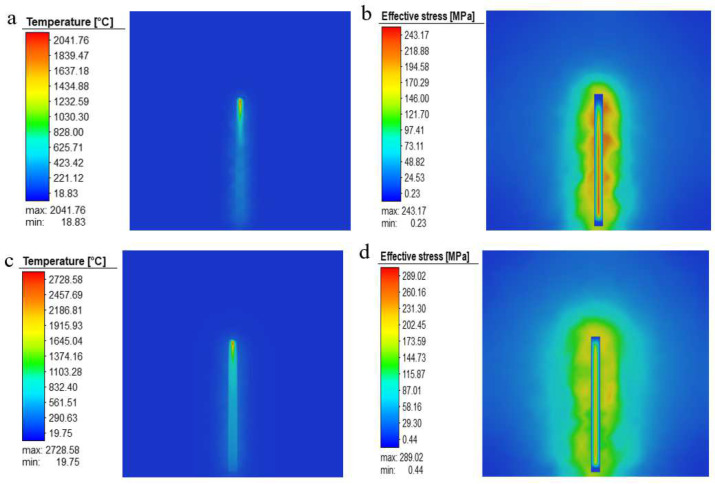

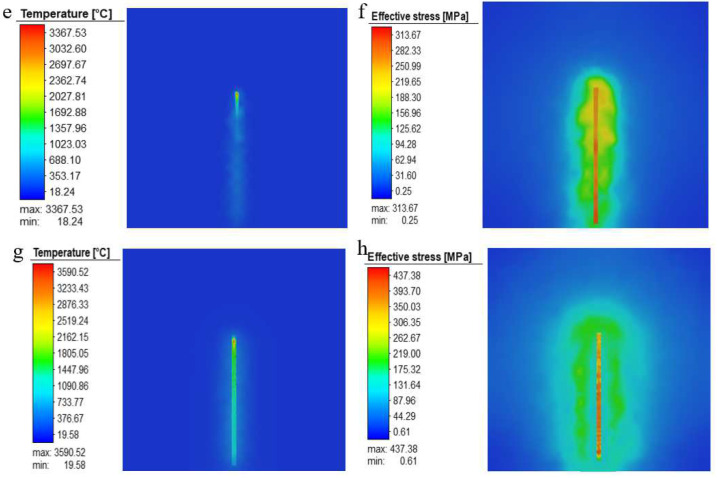
(**a**) Temperature field in layer 1 of experimental scheme 2, (**b**) stress field in layer 1 of experimental scheme 2, (**c**) temperature field in layer 10 of experimental scheme 2, (**d**) stress field in layer 10 of experimental scheme 2, (**e**) temperature field in layer 1 of experimental scheme 8, (**f**) stress field in layer 1 of experimental scheme 8, (**g**) temperature field in layer 10 of experimental scheme 8, and (**h**) stress field in layer 10 of experimental scheme 8.

The simulation results were selected to compare and analyze representative scenario 2 with fewer defects and scenario 8 with more defects. As can be seen from the temperature field diagram in Figure 12, the variation and distribution of temperature in the powder layer change dramatically with time and space. The shape of the radiation range of the heat source in the melt pool is similar to that of a comet, and it is more intuitive to see that the temperature distribution at the front of the melt pool is in great contrast to that at the rear, with a greater temperature gradient at the front of the laser beam. The reason for this phenomenon may be due to the fact that the heat transfer rate of the powder in front of the laser beam is much lower than the thermal conductivity of the solidification layer. In addition, comparing the melt pool temperatures of experimental solutions 2 and 8, we can see that as the energy density increases, the melt pool temperature increases significantly, but both show that the temperature when forming the first layer is significantly lower than in the tenth layer, and the heat-affected zone of the tenth layer is significantly wider. It is assumed that the reason for this is that the first layer of powder is connected to the substrate, which is at room temperature, and the thermal conductivity of the substrate is greater than that of the surrounding powder so that most of the laser energy input is absorbed by the substrate. As the new layer is formed, after the laser scan, the solidified portion below and the melt pool release heat into the surrounding area, preheating the surrounding powder and, thus, creating a temperature field, which expands as the energy density increases and more heat is released.

From the stress field diagrams in Figure 12, it can be seen that the stresses in both experimental solutions 2 and 8 are mainly concentrated on the substrate when forming the first layer, and the stresses are not uniformly distributed on the solidified layer; moreover, the stresses increase as the number of printed layers increases, at which point the stress distribution area is mainly concentrated in the welding channel and its adjacent area. This is because, during the solidification and cooling of the molten pool in the initial stage of construction, most of the heat is absorbed by the substrate, which causes the thermal expansion of the substrate and introduces residual stress. At the same time, the stress on the substrate reacts to the solidified layer. With the increase in the construction height, the effect of the residual stress introduced by uneven heating and cooling is more significant. In the part of the solidified layer with large and concentrated stress, the solidified layer cannot shrink freely, thus showing the phenomenon of uneven stress distribution in the solidified layer. As the final presentation of thermal stresses, the main determining factor for the occurrence of residual stresses in the solidification layer is still the large temperature gradients within the melt pool.

For program 8, where the defects are most severe, the main reason for the defects is that with the high energy density used for construction, the melt pool temperature also increases and the activity within the pool becomes more intense; this creates a huge temperature gradient with the surrounding area. The difference in the temperature gradient leads to a strong Marangoni effect being induced on the surface of the pool, causing the molten metal to be pulled from areas of low surface tension to areas of high surface tension, where cracks tend to form at the weld. In the case of larger cracks, due to the repeated thermal cycling of the laser, the cracks expand and contract frequently, and eventually, the cracks expand to form holes. In addition, the activity inside the melt pool causes the unmelted powder to enter the melt pool with the flow of the metal fluid. The sudden cold and hot forming characteristics of SLM allow the unmelted powder to be heated and melted in time, forming a molten residue that prevents the surface of the metal fluid from solidifying and forming a hole.

**Figure 13:**
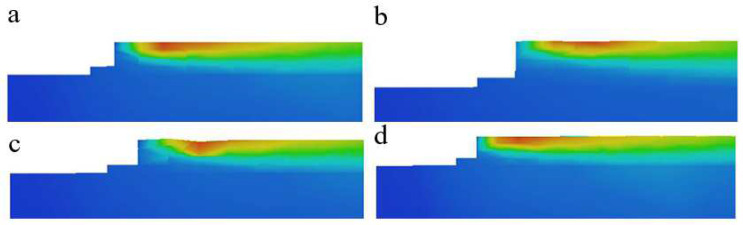
Cross-sectional view of SLM melt pool: (**a**) E = 83.3 J/mm^3^, (**b**) E = 41.7 J/mm^3^, (**c**) E = 111.1 J/mm^3^, and (**d**) E = 57.1 J/mm^3^.

In addition, it can be seen from the sectional view of the molten pool in Figure 13 that when the energy density is increased from 41.7 J/mm^3^ to 111.1 J/mm^3^, the change in the energy density has a greater impact on the solidified layer in the longitudinal range. Due to the deepening of the longitudinal heat-affected zone, the internal activity of the molten pool is more intense, which can easily cause the remelting of the solidified layer below. At the same time, due to the increased activity of the molten metal in the molten pool, it is very easy for the unmelted powder to enter the molten pool with the flow of liquid metal and solidify and adhere to the surface.

In summary, it can be concluded that the selection of suitable construction parameters for BCC-structured skeletal scaffolds can significantly reduce the generation of residual stresses, thus providing a reference for the process parameters for the application and preparation of bone scaffolds; on the other hand, the residual stress data obtained by means of numerical simulations are also a certain reference in terms of residual stresses in bone scaffolds, thus optimizing the process preparation parameters and enhancing the application and promotion value of the scaffolds.

### 4.5. Bone Scaffold Tissue Structure

There is still a gap between the simulation of the temperature field and the actual processing and forming. The experimental powder particle size, the absorption rate of the material, etc. can have an impact on the results of the finite element analysis, and the element migration and grain size in the melt pool are not accurately reflected. In order to better reflect the influence of different construction parameters on the forming quality of porous scaffolds, the present study analyzes the organization and size of the melt pool.

**Figure 14:**
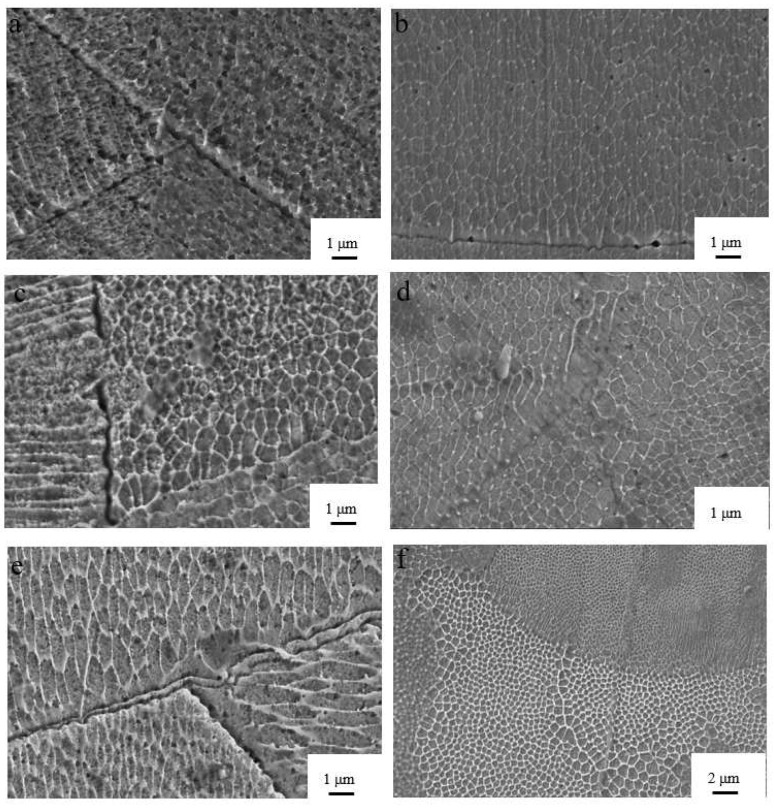
Scanning Electron Micrographs (SEM) of tissue structures at different energy densities: (**a**) E = 83.3 J/mm^3^, (**b**) E = 41.7 J/mm^3^, (**c**) E = 67.7 J/mm^3^, and (**d**) E = 100.0 J/mm^3^, (**e**) E = 111.1 J/mm^3^ (**f**) E = 57.1 J/mm^3^.

Figure 14 shows the tissue structure at different energy densities. From Figure 14, it can be seen that different areas of the melt pool take on different crystalline shapes, with cellular dendrites predominating near the edge of the melt pool and a typical equiaxed hexagonal cellular structure located away from the weld seam. The analysis found that the closer the melt pool boundary, the more the grain shape gradually elongated, and it eventually formed a strip of equiaxed crystal organization at the melt pool boundary, the reason for this situation is that the grains along the manufacturing direction of stretching and the new grains between the adjacent melt layer are subject to tensile-stress outward growth. The larger temperature gradient and the arc-shaped area of the melt pool also play a non-negligible role in the outward growth, the addition of new layers and the creation of the grains. The sudden cooling and heating of the SLM also leads to a strong Marangoni effect, which also affects the growth of the dendrites.

**Figure 15:**
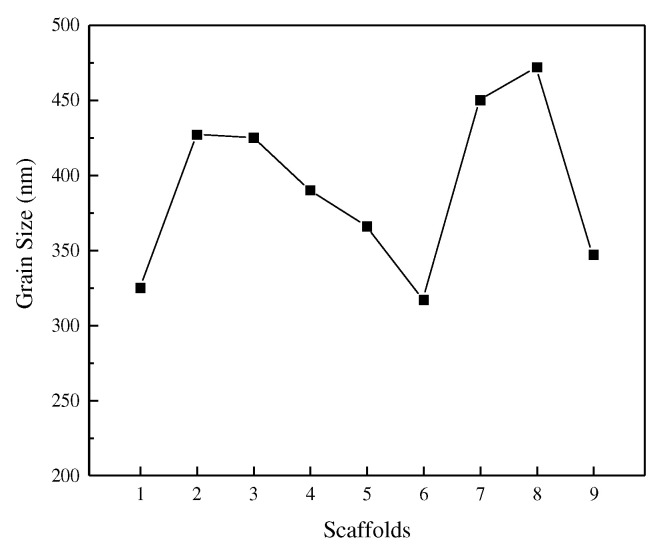
Effect of Energy Density on Grain Size.

Figure 15 shows the grain size for various process routes. Different energy densities create different temperature fields, and the graph shows that the energy density increases from 41.7 J/mm^3^, and then, shows an inverse relationship with grain size, with grain size gradually decreasing to a minimum of 321 nm at an energy density of 77.4 J/mm^3^. Figure 15 e,f also show that high-energy density processing forms an isometric hexagonal tissue grain and a more disorderly arrangement that is not compact; moreover, the edge of the weld is obvious and cracks are present. The reason is that in the case of higher energy density, the molten pool with rapid cooling and rapid heating changes significantly; the metal liquid produces violent surface tension phenomena, which then produce cracks, while a lot of repeated high-energy beam irradiation is absorbed and solidifies the layers. The repeated high energy exposure causes the solidified layer to absorb a large amount of heat to promote grain growth and deformation.

**Figure 16:**
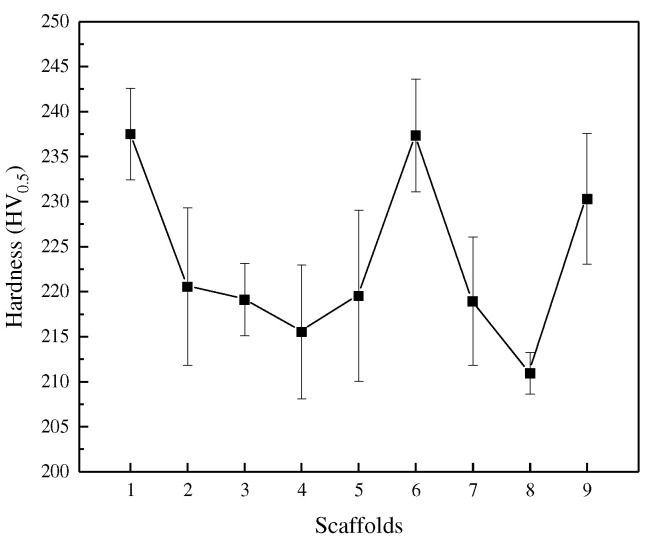
Hardness of the bone scaffolds.

The most intuitive effect of a change in grain size is the change in hardness, which, combined with the Hall–Petch equation, shows that as grain size decreases, the strength or hardness of the material changes as a function of the rising law. The hardness data were obtained using the indentation method, and Figure 16 shows the hardness of the optimized BCC scaffolds. From the results, it is clear that the presence of defects such as internal microscopic holes significantly reduces the hardness of the scaffold, with the hardness of the bone scaffold formed by process routes 7 and 8 showing the worst performance compared to the others.

The quality of the bone scaffolds and the presence of residual stresses can affect the plastic deformation of the pressed-in parts and, thus, make a difference to the hardness measurement. Of course, the presence of defects such as holes and cracks leads to a reduction in the forming density, which is also an important factor in the reduction in the hardness of the scaffold.

### 4.6. Residual Deformation of the Bone Scaffold

The main cause of process defects such as deformation and cracking in scaffold forming is caused by residual stresses during laser forming. Porous scaffolds are known as an ideal material for bone replacement due to their excellent structural customisability, but due to the presence of residual stresses, they are prone to different degrees of deformation, which, in turn, endangers their shape and dimensional tolerances. The use of different process parameters in the SLM forming process of the porous scaffold can easily affect the forming accuracy of the scaffold, so investigating the influence of different process parameters on the amount of scaffold deformation is necessary to improve the forming accuracy.

**Figure 17:**
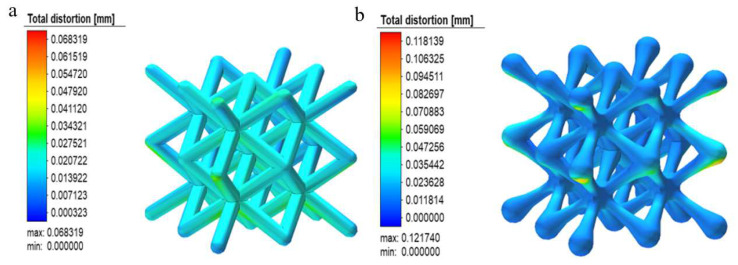

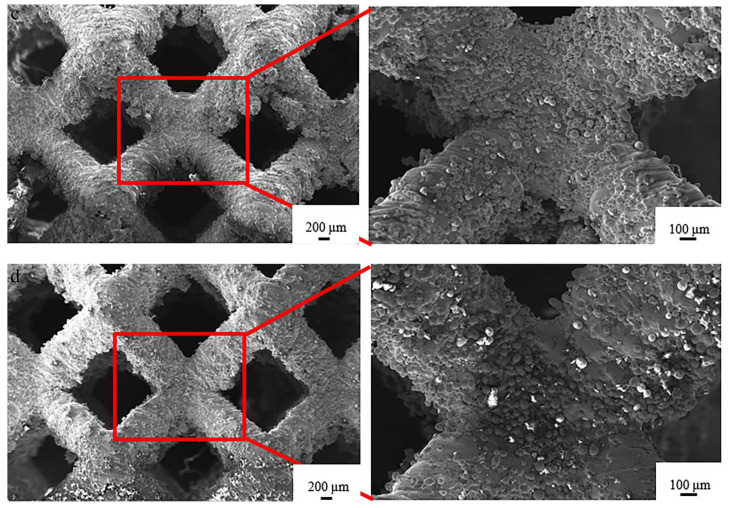
SEM photos of two kinds of bone scaffold: (**a**) finite element simulation of deformation of conventional BCC structure, (**b**) finite element simulation of deformation of optimized BCC structure, (**c**) surface morphology of conventional BCC structure, and (**d**) surface morphology of optimized BCC structure.

**Figure 18:**
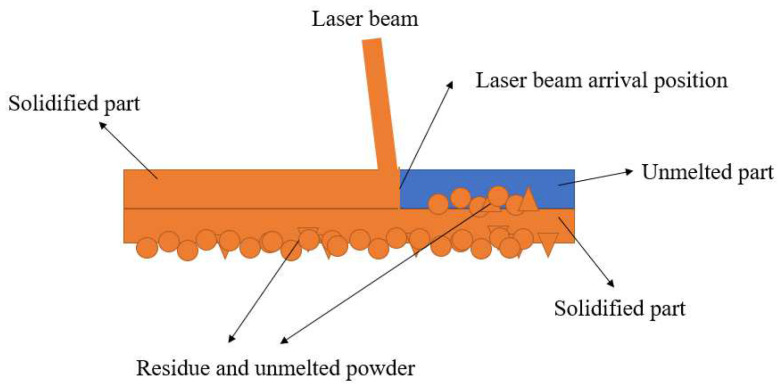
Schematic diagram of overhanging section additive manufacturing principle.

The dimensional accuracy of the bone scaffold is an important requirement for implant fixation and bone regeneration. Figure 17 shows a comparison of the deformation of the two structures, and it can be seen that there is a large difference in the deformation of the two structures. In the BCC structure, more obvious dimensional errors can be observed in the struts or nodes, and the deformation of the BCC structure increases after optimization, with a maximum deformation of 2.18%; this is, of course, related to the inclusion of the nodal ball. The deformation of the two types of scaffold is basically the same, with the part of the scaffold that is deformed being near the unit bars and below the connection points. There is a large amount of unfused 316L powder on the surface of both brackets, with a little more powder adhering to the surface of the conventional BCC structure. We can also see, in Figure 17, that the particles on the lower surface of the cell column show loose adhesion, while the upper surface has much stronger and smoother adhesion. On the one hand, the reason for the adherence of a large amount of unmelted powder may be that the spherical structure was suspended during the construction process, and when the nodal spherical section with fewer layers was constructed, the contraction force during the cooling phase was weakened by the lower layer of powder, resulting in increased contraction. The reasons for adhesion to the lower surface, on the other hand, can be analyzed in terms of the temperature field, as shown in the schematic diagram of overhanging section additive manufacturing forming in Figure 18. SLM forming uses a layer-by-layer cumulative thermal process where the new layer is always completely exposed to the laser beam and the powder is completely melted. Due to the poor heat transfer from the surrounding unmelted powder, the heat is mainly dissipated through the lower solidified layer. The unsupported lower surface at the bottom is in direct contact with the powder, which can easily lead to overheating, so when the melt pool solidifies, the unmelted powder adheres to the lower surface and increases the surface roughness. When the energy density is higher, the powder absorbs more energy and the increased fluidity of the melt pool causes some of the unmelted powder to enter the pool; when the laser beam is heated again, the unmelted powder slumps under gravity due to the unsupported bottom. The large amount of spheroidization on the surface can also lead to geometrical deviations in the unit column and, ultimately, to local stress concentrations.

It has been found that the surface orientation of a unitary column has a considerable effect on surface roughness and that a greater thermal gradient in a unitary column with a certain angle of inclination leads to slower heat transfer compared to a vertical strut; therefore, unmelted particles may adhere to the bottom surface, increasing roughness. However, a certain degree of surface roughness is biologically advantageous as surface roughness promotes osteocyte attachment to the scaffold surface for aggregation; however, greater roughness affects the fatigue properties of the implant, static strength, frictional behavior, fluid flow, and heat transfer, affecting the implantation effect.

In summary, it can be concluded that the finite element simulation combined with the theoretical model can make certain predictions on the actual scaffold forming surface quality, and the prediction results have a certain value. Using finite element simulation to make predictions on the experimental results in advance can improve the preparation efficiency, while significantly reducing the experimental time.

## 5. Conclusions and Future Prospects

In the process of SLM-technology forming, different structures or different processing parameters can easily affect the performance of the scaffolds to different degrees. After studying BCC-structured bone scaffold forming from the aspects of structure optimization and processing parameters, the main conclusions are as follows:

1. This study found that the addition of a nodal-ball transition to the conventional BCC structure junction significantly improved the strong resistance of the scaffold itself and reduced the phenomenon of toughness fracture; moreover, combined with the hydrodynamic data analysis, the improved structure did achieve good and desirable results in terms of permeability and enhanced cell aggregation.
2. It was found that the BCC structural bone scaffolds prepared with an energy density of 41.7 J/mm^3^ had lower residual stresses, fewer process defects, and excellent mechanical properties, while the laser power and scanning speed were found to have a strong correlation with the final forming quality for a given powder thickness, with the scanning spacing having a smaller effect.
3. According to the research results of finite elements combined with the experiments, it is suggested that when building BCC-structured bone scaffolds, if a high laser power is used, a larger scanning speed should be selected, and if a low laser power is used, a smaller scanning speed should be selected; this can prevent the expansion of the heat source radiation range, and reduce thermal deformation and the introduction of residual stress. In addition, this processing scheme can also reduce the agglomeration and adhesion of unmelted powder, reduce the surface roughness, and finally, achieve the purpose of improving the manufacturing quality.

This study focuses on the exploration of the mechanical properties and microstructure of BCC-structured scaffolds, and analyzes the advantages of the optimized structure and the effect of energy density on the performance of the scaffold. The optimal design of these structures is currently a hot topic of research, such as the optimization of stress concentration locations in this study, and the development of topologically optimized structures [34] and functional-gradient structures [35] by others. In addition, the present study derived the construction rules for the manufacture of bone scaffolds from a physical point of view, but the biological point of view is still lacking. The cellular activity of the scaffold surface and the regeneration of bone cells in biological clinical trials are a key part of the long-term in vivo functional study of bone scaffolds. The successful fabrication of metal-based porous bone implants has demonstrated their potential in additive manufacturing technology for medical applications, but a large number of clinical trials are still needed to validate their true functionality.

## Figures and Tables

**Table 1:** Orthogonal parameter design.

Number	Laser Power *P* (W)	Scanning Speed *V* (mm/s)	Scanning Spacing *S* (mm)	Energy Density *E* (J/mm^3^)
1	100	500	0.06	83.3
2	100	600	0.10	41.7
3	100	700	0.08	44.6
4	130	500	0.10	65.0
5	130	600	0.08	67.7
6	130	700	0.06	77.4
7	160	500	0.08	100.0
8	160	600	0.06	111.1
9	160	700	0.10	57.1

**Table 2:** Parameters of BCC structure model.

Structural Parameters	Cylindrical Diameter (mm)	Cylindrical Length (mm)	Unit Aperture (μm)
	0.5	4	850

**Table 3:** Optimization of BCC structure model parameters.

Structural Parameters	Cylindrical Diameter (mm)	Cylindrical Length (mm)	Nodal Ball Diameter (mm)	Corner Transition Radius (mm)
	0.4	4	0.8	0.6

**Table 4:** Comparison of compressive strength of two structures.

	Compressive Strength (MPa)	Compressive Strength of Human Bones (MPa)
**Conventional BCC Structure**	200	Cortical bone: 102.9–140.7 Cancellous bone: 3.2–7.8
**Optimized BCC Structure**	210	

**Table 5:** Modulus of elasticity of porous supports.

	Route	1	2	3	4	5	6	7	8	9
**Modulus of elasticity (GPa)**	**Conventional**	18.78	18.56	18.69	18.52	18.68	18.76	19.71	19.82	18.45
	**Optimized**	18.81	18.80	18.90	18.86	18.75	18.81	19.50	19.78	18.73

## Data Availability

The data presented in this study are available on request from the corresponding author.
